# The process of change for people with cognitive impairment in a residential rehabilitation program for substance problems: a phenomenographical analysis

**DOI:** 10.1186/s13011-019-0200-y

**Published:** 2019-03-29

**Authors:** Julaine Allan, Susan Collings, Alice Munro

**Affiliations:** 1 Lives Lived Well, 91 Dalton st, Orange, NSW 2800 Australia; 2 School of Education, University of Sydney, Sydney, Australia; 3 Western Local Health District, Orange, NSW Australia

**Keywords:** Cognitive impairment, Residential rehabilitation, Behavior change, Treatment programs

## Abstract

Cognitive impairment is prevalent among people with substance problems and a factor affecting retention in treatment. Empirical phenomenography was used to systematically explore how people with cognitive impairment viewed a novel residential rehabilitation program – Project RE PIN – designed with cognitive compensatory behaviour change activities and from a strengths-based approach. Twelve participants took part in semi-structured interviews and cross-case analysis identified the overarching theme of change. Key program elements were the safe environment, structured routines, modified psycho-educational material and staff support. Critical changes that participants attributed to the program were in dealing with their own and others’ emotions, experiencing daily life without drugs or alcohol and reframing their self-view. Fear and anxiety about relapse were common and few participants had strategies or support to cope in the future. This study demonstrates that program activities changed participants’ thoughts, feelings and behaviours about themselves and their substance use. The results indicate that RE-PIN’s modified content and processes can benefit people with cognitive impairments in treatment. The study highlights that some treatment users may be vulnerable to resumption of drug use despite gains made during a residential program and their desire to remain substance-free.

## Introduction

Drug and alcohol treatment interventions aim to change a regular harmful behaviour. Current approaches to drug and alcohol treatment predominantly use psycho-social methods, usually cognitive behaviour therapy (CBT), as an adjunct to medication within individual and group counselling and residential rehabilitation programs. Relapse after substance treatment is reported to be commonplace, with studies suggesting 94% of people have used at least once 12 months post-treatment [1] and between 40 and 60% of people returning to substance dependence [2, 3].

Behaviour change theories and relapse prevention models identify environmental triggers such as contact with other drug users and drug availability [4]; psycho-biological cravings [5], mood instability [1] and limited self-efficacy [6, 7] as important factors in high relapse rates. To minimise relapse, high intensity treatment options are recommended for severe and chronic substance dependence where reducing or abstaining from drug use is perceived to be more difficult [8, 9].

Residential rehabilitation is the most established treatment option for people with severe substance problems [10]. Treatment components typically include group and individual therapy, psycho-education, and within 12-Step and therapeutic community models - self-help and mutual aid groups. The most significant factors predicting success (abstinence or reduced drug use) following residential treatment were found to be treatment completion/retention [11, 12]; continuing care post-discharge; employment; and older age [13]. Improved retention is also associated with factors other than client characteristics. These factors include strong client-staff relationships, a supportive and comfortable environment, and a consistent daily routine [14–17].

Completion rates in residential rehabilitation vary widely (i.e. 9–75%), averaging approximately 30% [13]. Clients stay just one third of the time they planned to [11]. An association between substance use and cognitive impairment may partly explain why people with substance problems do not engage with residential treatment or leave early [14, 18]. Prevalence estimates of cognitive impairment among treatment-seeking-substance users vary from 30 to 80% [19, 20]. People with cognitive impairment are also less likely than others to complete treatment [14].

In their prevalence study, Allan et al. (2012) defined cognitive impairment as an umbrella term used to refer to the impacts of long-term drug or alcohol use, acquired or traumatic brain injury, intellectual disability, or Foetal Alcohol Spectrum Disorder (FASD) [21]. While each of these conditions can vary in severity and impact, they have similar broad effects on executive function [18, 22]. Individuals with some form of cognitive impairment will typically experience one or more of the following: impaired ability to plan and make decisions, reduced ability to evaluate consequences, a preference for reward-seeking goals, impulsivity and attentional dysfunction, lack of initiative, memory deficits, impaired self-monitoring and self-regulation, and an inability to benefit from experience [18, 21]. Further, those with some form of cognitive impairment are more likely to experience poor concentration, depression, emotional instability, irritability, impulsive or inappropriate behaviour, reduced ability to problem-solve and inflexible thinking [23, 24]. These factors suggest that people with cognitive impairment are likely to have difficulty engaging with and participating in substance misuse treatment that is predominantly based on cognitive and behavioural change activities.

In recognition of these issues and driven by the imperative to improve treatment retention of people with cognitive impairment, a program entitled ‘Project RE PIN’ (Receive, Encode, Process and Integrate drug and alcohol treatment strategies for people with cognitive impairment) [hereafter RE PIN] was developed. To facilitate this novel approach to practice change, the organisation secured philanthropic funding for a project coordinator to synthesize existing evidence of inclusive program models that were suitable for the rehabilitation context and for an independent evaluation of program development.

The RE PIN program aimed to enhance the lives of individuals with cognitive impairment and substance problems, by developing, implementing and evaluating a new type of drug and alcohol rehabilitation that was inclusive of people with cognitive impairment. The program was developed using strengths-based principles and person-centered practice because these approaches had been shown to improve treatment retention in some studies [25, 26]. The program content was devised using universal design principles to ensure suitability for people with cognitive impairment. Universal design endeavours to make environments, resources and education methods accessible for people with CI [27, 28]. Psycho-educational groups, program materials and activities were designed to be easily understood: the provision of information and skills was delivered in ways that met a variety of learning styles; and intentional strategies for people who needed assistance to understand and retain routines, tasks and instructions were used. The psycho-social components focused on practicing skills before introducing concepts, used repetition and role play, and simplified written material to complement verbal instruction. Daily routines were strictly maintained, residents were assisted to use memory aids such as diaries, and staff were trained to understand how cognitive impairment could manifest in behaviour and how to use simple inclusive techniques such as reminders about appointments, tasks or house rules, rather than interpreting forgetfulness as non-compliance.

### Aim

This study aims to understand how a residential substance use rehabilitation program designed to be inclusive of people with cognitive impairment influenced the treatment experience of residents with cognitive impairment. The study was part of a larger project to document the process of developing and implementing RE PIN that sought the perspectives of residents, staff and management.

## Method

### Ethics

Ethics approval to conduct the research was granted by the UNSW Human Research Ethics Committee in April 2016 (reference number: HC16131). Program materials available from first author on request.

### Study context

Wattlegrove is a 3-month voluntary rehabilitation service for men and women located in regional NSW, Australia. It works with up to 16 people at a time after each resident completes 1–2 weeks of medically supervised withdrawal prior to commencement. A previous study has identified that almost half of Wattlegrove’s residents were likely to have a cognitive impairment and that program completion rates for this group were significantly lower than for residents without a cognitive impairment (10 and 56% respectively) [22]. As a result, in 2016, Wattlegrove was modified to better meet the needs of people with a cognitive impairment while maintaining its ability to treat those without cognitive impairment. A strengths-based approach was used to shape the RE PIN program [25, 26]. Strengths and skills intended to be developed by the program included self-determination, empowerment, choice and control in daily functioning [29–31]. The program ethos was guided by the Virtues Project [32], a United Nations supported anti-violence program, and modelled by staff to encourage self-reflection, boundary setting and enhanced self-esteem.

### Study design

Empirical phenomenography was used to systematically explore participants’ experiences of the treatment program. Phenomenography is designed to identify the similarities and differences in participants’ descriptions of the same complex social phenomenon [33]. The phenomenon in this study was a residential rehabilitation program in which all residents participated in the same routines and activities including psycho-educational and therapeutic groups. The theoretical basis and delivery modalities of the program were not explicitly explained to participants, so their perspectives were constituted by their own understanding of the phenomenon [34].

### Participants and data collection

Sixty-seven people took part in the rehabilitation program during the 12-month study period of which 33 were assessed as having a cognitive impairment. Prior to their admission, all residents completed a 7–14-day withdrawal program and were assessed by a medical practitioner as having completed withdrawal. A member of the research team (Author: AM) administered a cognitive screening tool, Addenbrooke’s Cognitive Examination Revised (ACE-R) to all residents during their first week in the program to determine eligibility to participate in the evaluation. The ACE-R is a brief cognitive screening tool that has been used to detect cognitive decline and monitoring cognitive function [35]. Although the ACE-R was designed for the dementia field, it, like the Montreal Cognitive Assessment (MOCA), has been used in many other populations and has an Australian version [36, 37].

All eligible residents consented to participate in the study and were invited to an interview to occur eight to ten weeks later, to allow enough time for them to reflect on their experience of the program. Eighteen people agreed to an interview but six had left the program by the interview stage and could not be contacted. A total of 12 residents took part in an interview conducted by a member of the research team (Author: SC). Semi-structured, open-ended interviews took place via computer video link between July 2016–January 2017. Participants were asked to reconfirm consent prior to their interview and consent to record the interview was also provided. Interview topics included: how the person came to be in the program; how well it met their expectations; positive/less positive aspects; how helpful it had been; plans/concerns for the future.

#### Data analysis

**Fig. 1:**
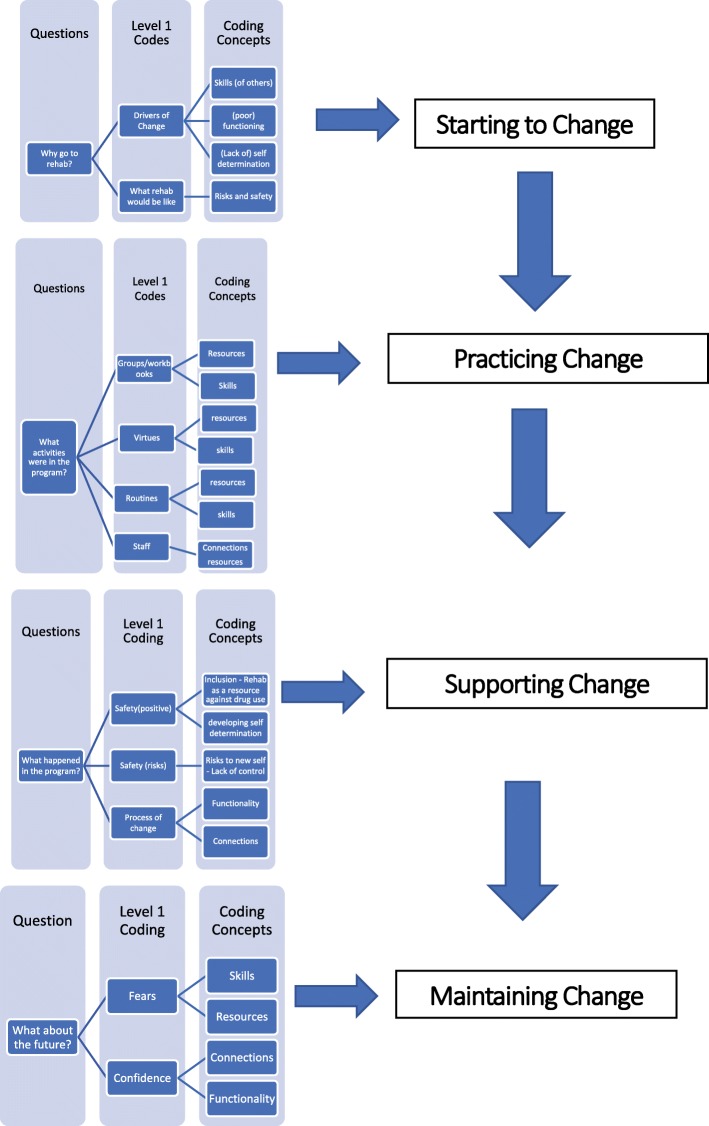
Summary of codes and concepts

The interview transcripts were deductively coded (Author: JA) using the interview schedule to sort the transcripts into initial codes based on the answers to the interview questions (Fig. 1). NVivo software (QSR International Pty Ltd) was used for data management. Systematic text condensation (STC) was used to organise and code the interview data into concepts by comparing the content and meaning of responses [38, 39]. STC is an appropriate method for a small sample describing the same thing [40]. Initial codes were reviewed and participant responses sorted into descriptions of their experiences for each question and the text was read line by line to identify elements of the coding concepts listed in Fig. 1.

The concepts of interest were determined by the strengths-based philosophy of the rehabilitation program as being personal change in perceived substance use and how this change was related to the program elements of resources, connections, skills, empowerment and functionality in daily living [30, 40]. Additional concepts of risk and safety were identified during the coding process and incorporated into the analysis. Co-authors (SC, AM) reviewed the codes and concepts to confirm validity during the analysis process. Once coded, the re-organised pieces of text were read and analysed according to the key phenomenographic concept of interest: the influence of the inclusive rehabilitation program on participants’ experience of treatment.

The final cross-case analysis identified the overarching theme of change as the coherent concept grounded in the participants’ accounts of the residential rehabilitation experience [38]. Findings are organized by starting to change, practicing change, supporting change, and how change may or may not be maintained in the future.

## Results

### Characteristics of all screened residents (*N* = 67)

There were similarities and differences between residents with cognitive impairment (*n =* 33) and those without (*n =* 34). Both groups were similar in terms of age, gender and Indigenous status; however, they differed in the main drug of concern. Amphetamine was the main drug of concern for residents with cognitive impairment (50% compared to alcohol 28%) whereas alcohol was the main drug of concern for residents without cognitive impairment (47% compared to amphetamine 35%). Residents with cognitive impairment were more likely to be younger and to have left school by or before 16 years compared to residents without cognitive impairment. Retention rates were similar across both groups. Residents with cognitive impairment were more likely to complete the program than those without (49 to 41%). However, this was not statistically significant. Almost half of all residents did not complete the program and there was no difference for those with cognitive impairment (47%) and those without (48%). Characteristics of study participants (*n* = 12).

Study participants were, on average, 34.5 years and had an ACE-R score of 77.5 (range = 63–85^1^). All participants relied on government payments for income. Alcohol was identified as the primary drug of concern by five participants (42%), three identified cannabis (25%), three identified amphetamines (25%) and one person identified opioids as their principal drug of concern (Table 1).

**Table 1:** Characteristics of interview participants (*n* = 12)

No.	Sex	Age	ACE-R score	Primary drug of concern	Age left education	Income
1	F	32	82	Amphetamine	15	Temporary benefit
2	M	25	63	Cannabis	18	Disability benefit
3	M	32	70	Amphetamine	16	Temporary benefit
4	M	25	80	Cannabis	15	Temporary benefit
5	M	33	82	Amphetamine	15	Disability benefit
6	M	23	80	Alcohol	16	Temporary benefit
7	M	26	80	Opioids	15	Temporary benefit
8	M	43	78	Alcohol	22	Temporary benefit
9	F	33	71	Alcohol	16	Temporary benefit
10	M	19	76	Cannabis	14	Disability benefit
11	M	59	84	Alcohol	16	Temporary benefit
12	F	65	85	Alcohol	14	Temporary benefit

### Starting to change

Most participants reported being required to enter the rehabilitation program due to consequences of their drug use including incarceration, loss of legal access to children or family breakdown, and no one said they had the decision had been driven by a wish to improve physical or mental health problems. While the program was voluntary, drivers of change were legal systems, as summed up by Participant 11: “*I was on parole. I’m on parole now. I messed up. I drink drove and got caught.”* Family breakdown and relationships were frequently mentioned, as Participant 3 stated: *“I haven’t seen my kids for two years, and for me to get them back into my life, I knew I had to fix myself up.”*

Several participants referred to the role of unspecified others, usually described as ‘they’, who were likely to be health or community services staff, for their entry to treatment. As Participant 9 explained “*Yeah, I’ve had about five attempts at detoxing and it didn’t work. They said I have to do rehab, so I agreed.”* Agencies were a significant pathway to rehabilitation. For example, as Participant 6 stated:

*“They said that it would be good for me to come to rehab and I said ‘Well, if I can get in pretty much straight away. I would rather do it now’. I didn’t really know what to expect. I didn’t really know what we would be doing there and stuff like that.”*

Half of the participants had not been to a residential rehabilitation program previously and described uncertainty and apprehension. Participant 3 said: “*I was told that rehabs were full of drugs as much as what’s out in the street. So, I thought that wasn’t for me. I didn’t really know what it would be like,*” and Participant 9 reflected on concerns about the people she would encounter, saying *“I thought it would be full of angry people and a bad environment.”* However, no one questioned that rehabilitation could help change substance use problems or expressed doubt about its potential effectiveness. As Participant 1 stated: *“I’ve come through a rough time, drug and alcohol stuff. I thought it was time for a change, so they got me in.”*

While most participants were apprehensive about entering residential rehabilitation, this was clearly perceived as preferable to not going. Participant 4 noted: “*I was too scared to go back out in the world* [after the supervised withdrawal program].” All but one participant chose to enter treatment freely because they thought it would reduce their drug use. Participant 7 expressed resistance to entering the program and indicated he felt forced to enter:

*“To tell you the truth, I was in detox and I wanted to go home. Then they hounded me and hounded me to go to rehab. I had to ring 100 different rehabs and then she was like, ‘We got you a bed’ and I thought, ‘My gosh, now I'm going to have to be there.’”*

### Practicing change

Four program elements were described by all the participants as assisting them to develop skills, resources and connections. The four elements were group-based and included use of workbooks, daily virtues, routines, and staff.

#### Groups

Most participants described the psycho-educational groups as helpful with understanding personal triggers for substance use, practicing strategies to manage these triggers, and coping with cravings. Participant 4 connected her emotional responses to situations with drug use triggers:

*“I think, what I'll take away is to understand that that's the person I am, and I'll manage it. To understand my feelings, like when I am angry, to get away from the situation and take a breath and understand my feelings, I guess. Just understand what I'm feeling. If I'm angry, I know there's other options than to go use, or drink.*”

*Participant* 11 described connecting to the group process and content:

*“I found it’s a good pace. They slow it down and break everything down. They don’t want you to miss nothing. You absorb it all and in the end of it, you’re coming out with these answers that are deep inside that you didn’t know you had.*”

Most people reported that the workbooks designed to supplement group work material assisted with revision and reflection. Several participants said the content resonated with their experiences. For example, Participant 9 stated

*“The book I’m on now, it’s trying to say how you can say no to drugs and alcohol without offending people. Like, don’t be aggressive; be assertive. That type of thing. It really makes you think. When I was in my prime of alcoholism, I just never thought of those things before.*”

The way participants felt about their own abilities affected their views of activities. For example, some were comfortable seeking help from others: “*It’s really good. It’s easy to ask for help here. You don’t feel like you’re dumb, I guess. They just help you.*” [Participant 5] or found the material and the support well-matched to their needs:

*“I’m not 100 percent there in the brain, but I found it completely at my level and, if I did struggle, I just had to put my hand up and say maybe explain it a different way, but I didn’t get anxiety or stressed over it.*” [Participant 2]

Others described the opposite, demonstrating that the groups and workbooks were not suitable for everyone. For example, Participant 9 felt unable to use the materials or ask for help:

*“Maybe a bit more one-on-one support would be good for me, during groups and IP* [Individual Program – goal setting and action planning]*. Some things they talk about go straight over my head and I don’t want to ask in group, because I feel embarrassed.*”

Feelings of shame or embarrassment affected people’s ability to use the resources in the ways they were intended regardless of availability of staff assistance. This is summed by Participant 3 who said, “*I don’t want the other people to think I can’t do anything*.”

#### Virtues

The virtues program was a self-esteem development program that involved participants describing themselves positively and encouraged respectful interactions between residents. Participant 3 explains the process and the purpose of the virtues:

*“We've got virtue cards and the virtue is basically explained on the card, front and back. Kindness explains how to be kind to people. It's pretty easy, and if you don't know what they are, you just google them. We go through them every day. There's a 100-card pack and 100-odd virtues. So, you memorise one for the day and talk about a way you can practice it that day. Today's virtue was commitment, and basically, commitment to me is just being committed to being human and to my family.*”

Eleven of the twelve study participants specifically identified the virtues program as a positive program element. The virtues program was viewed to help build skills to understand self and others. Participant 1 described how she applied the virtues:

*“Every second of the day you're thinking of things that are relating back to the virtue and it just helps out so much to touch base with other people and even yourself.*”

Several participants described the empowerment they gained from improved self-appraisal, attributed to the use of daily virtues. Participant 12 explained:

*“The virtues, for one, they’re a great way to put things and reflect on and teaching me how to respond and not react to things, and just basically give myself a pat on the back and be proud of myself.*”

Some participants described using the daily virtues as a practical guide for daily interactions. For example, as Participant 10 put it:

*“Yeah, we read out the day’s virtue, whether it be caring, honesty or whatever. It’s good. I do like it. Every virtue, you think how you can use it. ‘How can I be helpful today?’ Just try and work on it, even if you can only help someone once. It’s a bonus for me.*”

Virtues were also seen as a resource to create a new, improved view of themselves. Participant 12 described interpreting herself differently through the words used in the virtues cards:

*“… so it makes me look at myself and I’ve put down things like I’m forgiving and humility, and really looking at me and going, okay, well, I’m not such a crap person, because I’m an addict. I’ve got some good values there.*”

Participant 11 described how the virtues program connected to other program materials:

*“I find the books really good, actually, too. A lot of it ties in with the virtues and they’re sort of bracketed down to how you’d deal with a situation or how you’re feeling*.”

Only one participant reported not wanting to participate in the virtues group and perceived no benefit from the activity; “*So, I don’t think virtues really work*.” [Participant 6].

#### Daily and weekly routines

All participants talked how integral that daily and weekly routines were. For example, Participant 2 described the morning routine:

*“We had morning chores [then] virtues group after breakfast then … our morning walk and we get ready for 10.00 classes. We had time to ourselves to use our computers or any outstanding matters [and] to get our personal stuff done as well.*”

Participant 3 described the afternoon routine and some of the weekly events:

*“You come back up to the rehab for lunch. Mondays, Wednesdays and Fridays, we go to the gym after lunch. Tuesdays and Thursdays, we do drumming. Dinner at 6.00, then we have another evening group around 6.30 or 7.00, which is basically the same as the morning group. You give your number, you reflect on the virtue of the day and everyone tells you how they're feeling. If I was an eight this morning and a nine tonight, I'd tell them why I've gone up. That's basically the day. On Fridays, they take us shopping at 10.00 for a couple of hours.*”

Most participants described the routine as offering “*consistency*” [8], *“security”* when new people came into the group [1] and using free time without drugs. Participant 7 said, *“I think we just learn to be bored, if that makes sense. Learn to figure out what to do when we’re bored without using drugs.*”

However, not all elements of the routine were viewed positively. Outings were part of the routine that felt unsafe for some people who wanted to avoid exposure to triggers. Participant 8 described feeling at risk; *“I don’t like a lot of outings because there’s a lot of triggers when you go outside the house*.”

#### Staff

Staff members were responsible for managing daily routines, delivering programs, and supporting residents with future housing, children’s visits, medical appointments and other tasks. Most participants viewed the staff as a resource. Participant 12 described staff as external support for emotional states such as *“running rough”* and Participant 3 identified continuity and communication between staff as important, saying *“They keep an eye on the notes and stuff from the shift changes. I’m guessing that’s how they know … when our moods change.*”

Staff were also responsible for facilitating residents’ participation in house processes to develop their skills in communication and decision-making. For example, Participant 6 talked about how residents were included in program implementation:

*“They always let us know if we had problems, we could go to someone to talk about things or if we wanted to put a complaint in about something we could put a complaint in. We always had that support there.*”

### Supporting change

#### Safety

Safety was a commonly described in relation to program aspects that helped or hindered their rehabilitation experience and was integral to change. The physical environment promoted safety, being secure and drug free, and some participants felt daunted by the prospect of returning to life outside. Participant 2 felt that the world beyond the house compromised his safety, saying *“I try not to go on the outings, because I don’t feel safe within my own self at the moment.*”

A sense of safety was created for new residents through interactions with other residents and staff. Participant 1 talked about how residents supported each other:

*“We've got a mixed group of young and old and some of the older fellows come in and it might be a bit too much for them. You just take a minute and whether they're having a cigarette or reading a book, you say 'You'll get used to it, it'll get better for you.' They come around.*”

Participant 3 noted that staff fostered a sense of safety by being attuned to people’s moods:

*“I've had down days and they're [staff] pretty quick to pick it up. I'm not the sort of person that likes to talk about emotions and let it out, but they're pretty quick. The times I've been down, they pick it up pretty quick.*”

Participant 4 referred to the safety of the physical environment saying,*” I love my room, it’s my space. I am safe there*.”

However, some interactions presented safety risks, in particular the risk of drug use. Participant 12 explained how one person can affect a whole group and the vulnerability to triggers:

*“There was a past resident that came back to the house, but while she was at the house, she was showing us no respect. She was on her mobile phone all the time and, basically, in the end, she was showing us pictures of drugs and stuff. We all had to call a house meeting of residents and say, ‘Look, this is what she’s doing. This is unfair.’ She was using, we could see that she was using, and, behind closed doors, she was showing us pictures of drugs, which triggered three of us to the point where we were really rough.*”

Importantly, Participant 12’s description of acting to address the safety risks demonstrates exercising empowerment and communication skills; “*We had to call a house meeting and get her out*.”

#### Process of change

Few participants spoke explicitly about the physical environment of the residential unit and ‘rehab’ was described as a process. For example, Participant 6 said that rehab; “*really makes you think. When I was in my prime of alcoholism, I just never thought of those things before.*” The process of change occurred over many weeks. Most participants did not know what was going to happen when they entered the program or how change would eventuate. Participant 4 explained *“You don’t realise until you start getting further on in the program how far you’ve come, how much it all helps*.”

Several participants referred to the unexpected changes of being drug and alcohol-free for eight to ten weeks. Participant 6 stated; *“There was no drugs or alcohol involved and pretty much the first time since I was a young teenager, I realised you can be happy. I don’t know. It was just a bit of a change in life.*” Participant 7 was surprised to find that life without substances could be enjoyable, saying *“we’d sit around laughing our heads off and actually we’d say we’ve probably never laughed so much in our lives. We were just sitting around with no alcohol, no drugs and just making do with what we’ve got.*”

### Maintaining change in the future

Many participants described the significance of interpersonal communication and connections in making changes. Several were conscious that maintaining these positive changes would depend on how they responded to others. For example, Participant 4 described a strategy to manage strong reactions:

*“I think, what I'll take away is to understand that that's the person I am and I'll manage it. To understand my feelings, like when I am angry, to get away from the situation and take a breath and understand my feelings, I guess. Just understand what I'm feeling. If I'm angry, I know there's other options than to go use, or drink. I guess, also like I said, understanding other people's differences.”*

Few participants had informal support networks to help them maintain changes. Participant 9 was one of the few who could identify family members saying, *“Mum said she’d make sure she’d keep an eye on me and just give me soft drink and make sure I stay away from the alcohol.*” Others had limited social networks and were nervous about reconnecting with friends who may pose a risk to them maintaining change. Participant 5 knew that friends would not change their behaviour to support him:

*“Not smoking pot is going to be the hardest part because a lot of my friends do it if I go into their houses and that. They’re not the sort of people who would say, “Go on, have one,” but just being around them while they’re doing it is going to be a trigger.*”

Whereas Participant 3 feared his prior social networks would encourage drug use:

*“That's going to be the hardest thing for me, seeing old mates and them asking if I want some. That's the hardest part. You are who you hang around. It's sad to say, but I've started hanging around some pretty ordinary people. You think they're your friends but they’re not.*”

After two months in the program most residents went home or stayed with friends or family for a week to prepare for leaving the program, which was known as ‘going on prac’. Participant 2 explained that, *“You plan your outside life and what you’re going to do or start planning your exit from there.*”

Returning home allowed people to test their new skills and identify any gaps or problems that needed further assistance. Participant 11 described it as a learning experience saying, *“Going with what they’ve taught you, like being with positive people, not placing yourself in a risky environment, using different virtues.*”

‘Prac’ generated fear and anxiety for some participants. For example, Participant 3 feared relapse:

*“I'm getting a bit anxious, knowing that I'm going. I've been here, wrapped in cotton wool for two months, and being released back into the big, wide world, I'm scared that I'm going to relapse.*”

Some participants did indeed relapse on ‘prac’ and were supported to try again. As Participant 7 explained, *“I did stuff up the first prac. My mum came down on holidays. I hadn’t seen her for three years so I drank. I had to go out on another prac.*”

Even if ‘prac’ went well, there remained concerns about relapse after the program finished. Participants had heard about or witnessed relapse by others after leaving. Participant 11 noted:

*“The other day, a guy relapsed. We were out shopping and we saw him, and I was a bit upset. He reached out for help and back out into the big, wide world he relapsed. So, obviously, he'll be using now.*”

Some participants were less confident about their own safety, especially if they had relapsed after previous rehabilitation programs. Only one participant was confident about his ability to stay abstinent and noted the importance of changing the environment:

*“I will be right. Sticking to a plan and a routine. Having a plan. Just doing those things that I’ve learnt in here. I just look at things a bit differently about maybe relocating somewhere else. Somewhere to get a fresh start.*” [Participant 6]

No one raised strategies for managing or reducing the risk of relapse or formal support or aftercare following discharge. Only Participant 8 mentioned ongoing support from staff saying, *“I can ring up. Some of the others, the past ones do it. The staff want to know what’s happening with people.*”

Most participants described significance of the changes achieved with one person summing it up as*, “As a matter of fact, it’s given me a second life.*” [Participant 2]. However, they lacked a sense of agency, control or confidence in being able to maintain the changes. Participant 3 explained there was just hope it would *“stick”* in spite of the risks, triggers and fears about returning to regular drug use.

## Discussion

This study explored how people with cognitive impairments experienced a novel residential rehabilitation program purposely designed to offer a strengths-based and cognitively compensatory methods for behaviour change. Twelve residents screened as having cognitive impairment took part in semi-structured interviews. Thematic analysis discerned change as the overarching concept that organised their experience of rehabilitation. Overall, the program met the participants’ needs for learning about and practicing behaviour change. The program was well received, relevant and liked by the study participants. This is a significant achievement when most of the participants did not voluntarily choose to enter residential rehabilitation but felt coerced or obliged to do so.

The environment was generally viewed as positive and supportive and the program content was accessible to people with cognitive deficits. People with cognitive impairment completed treatment at the same rate as others in the program (49%), suggesting the RE PIN program met their needs equally well. This is a substantial improvement on the retention rate prior to the program introduction, where only 10% of residents with cognitive impairment completed treatment [22]. However, strategies for maintaining change in the future and a lack of ongoing support were a source of fear and anxiety for the participants, suggesting a high risk of relapse.

Participants were clear about the environmental and personal triggers that posed a risk of ongoing drug and alcohol use. However, there was less clarity about how treatment could help upon entry to the program. Participants had implicit trust that the treatment program would help reduce their problematic substance use and program philosophy, strategies and techniques were not questioned. This degree of trust puts a significant responsibility on treatment providers to deliver something that works to meet people’s diverse needs and circumstances.

Being in a secure rehabilitation environment was perceived as a key safety factor and when that setting was breached by other residents’ drug use or by external factors such as outings, participants described a sense of vulnerability to resumption of drug use. Many expressed fear and anxiety about relapse and concerns about their ability to resist cravings. Drug availability combined with limited self-efficacy are critical factors in relapse and challenges for all those leaving a residential setting [4, 6, 7]. However, this group of residents were likely to be facing these challenges without identifiable sources of support, leaving them to rely on personal willpower to avoid or resist drug use. Aftercare has been identified as an important treatment component that assists in preventing relapse [13] and this did not appear to be available to participants in this study, or they were unaware of these options. Going on ‘prac’ was a test of self-efficacy but participants were aware that triggers and cravings for drug and alcohol use would persist for an extended period, perhaps indefinitely.

The program achieved its aim to deliver a strengths-based approach. Program content and materials were relevant, accessible and applicable to the rehabilitation needs of residents with cognitive impairment. This study confirms earlier research about the benefits of developing skills in communication, reflection, and self-awareness of the impact of moods on triggers to drug use [1]. There were multiple examples of plans to apply program learnings in the future and the daily virtues program was frequently cited as a practical way to build self-esteem and confirm personal values. Staff are established as being a vital component of successful treatment programs and provide support, security and stability for the group [15]. However, there continues to be a need for individualised attention within the group program to overcome shame or embarrassment about disability and ensure that the learning material is accessible for everyone. More individual planning could facilitate greater development and application of behaviour change strategies as well as post-treatment community reintegration in a safer fashion.

## Limitations

The results of this descriptive study are specific to the program and participants and therefore cannot be generalized to all residential drug and alcohol treatment. However, the experiences of these participants highlight the changes people experience in a residential program, how they perceive the program content and ways it can be applied. Further research on program outcomes through the administration of standardised instruments at commencement, completion, and 3 months post-treatment would further demonstrate the impact of the program on participants including those without cognitive impairments. Comparing the experiences of people with and without cognitive impairments could clarify which program components have the most impact. More research to establish an evidence-base for best practice in drug and alcohol treatment by comparing resident outcomes attained in this program to those found in conventional residential drug and alcohol treatment programs would be useful for the treatment sector.

## Conclusion

This study has explored the experiences of people with cognitive impairment in a novel residential substance treatment program. It has demonstrated ways that strengths-based program activities impacted on participants’ self-view and their substance use behaviours. The results describe the way people with cognitive impairment make use of treatment activities and derive benefit from treatment. However, further research is needed to understand how these benefits occur and how they can be maintained. The study also highlighted a vulnerability to relapse post-treatment despite clear intention to remain substance-free and the need for aftercare, particularly for those who lack social support.

## Footnotes

1 ACE-R upper limit score for cognitive impairment is 88
